# Contraceptive self-injection through routine service delivery: Experiences of Ugandan women in the public health system

**DOI:** 10.3389/fgwh.2022.911107

**Published:** 2022-08-18

**Authors:** Jane Cover, Allen Namagembe, Chloe Morozoff, Justine Tumusiime, Damalie Nsangi, Jen Kidwell Drake

**Affiliations:** ^1^PATH, Seattle, WA, United States; ^2^PATH, Kampala, Uganda

**Keywords:** self-injection, DMPA-SC, injectable contraception, family planning, Uganda, self-administration, depot medroxyprogesterone acetate

## Abstract

Contraceptive self-injection (SI) is a new self-care practice with potential to transform women's family planning access by putting a popular method, injectable contraception, directly into the hands of users. Research shows that SI is feasible and acceptable; evidence regarding how to design and implement SI programs under real-world conditions is still needed. This evaluation examined women's experiences when self-injection of subcutaneous depot medroxyprogesterone acetate (DMPA-SC) was introduced in Uganda alongside other contraceptive options in the context of informed choice. We conducted structured survey interviews with 958 randomly selected SI clients trained in three districts in 2019. SI clients demonstrated their injection technique on a model to permit an assessment of injection proficiency. A randomly selected subset of 200 were re-interviewed 10–17 months post-training to understand resupply experiences, waste disposal practices and continuation. Finally, we conducted survey interviews with a random sample of 200 clients who participated in training but declined to self-inject. Data were analyzed using Stata IC/14.2. Differences between groups were measured using chi square and *t*-tests. Multivariate analyses predicting injection proficiency and SI adoption employed mixed effects logistic regression. Nearly three quarters of SI clients (73%) were able to demonstrate injection proficiency without additional instruction from a provider. Years of education, having received a complete training, practicing, and taking home a job aid were associated with higher odds of proficiency. Self-reported satisfaction and continuation were high, with 93% reinjecting independently 3 months post-training. However, a substantial share of those trained opted not to self-inject. Being single, having a partner supportive of family planning use, training with a job aid, practicing, witnessing a demonstration and exposure to a full training were associated with higher odds of becoming an SI client; conversely, those trained in a group had reduced odds of becoming an SI client. The self-care program was successful for the majority of women who became self-injectors, enabling most women to demonstrate SI proficiency. Nearly all those who opted to self-inject reinjected independently, and the majority continued self-injecting for at least 1 year. Additional research should identify strategies to facilitate adoption by women who wish to self-inject but face challenges.

## Introduction

Over the past 5 years, the concept of self-care, defined by the WHO as “the ability of individuals, families and communities to promote health, prevent disease, maintain health, and to cope with illness and disability with or without the support of a healthcare provider (1)” has taken the reproductive health community by storm. There is widespread enthusiasm for the idea that, through self-care, women will overcome persistent challenges accessing health services, practices, and products that will improve their health while promoting greater reproductive autonomy. The COVID pandemic has further accelerated efforts to expand self-care to reduce burdens on strained health systems and ensure that women can obtain the products they need without risking COVID infection (2). Enthusiasm for self-care is expressed in the WHO Consolidated Guideline for Self-care Interventions for Health, which identified self-care as “one of the most promising and exciting new approaches to improve health and well-being (3).” While myriad research studies have shown the potential of self-care to improve access and health (4–10), demonstrating the “why” of self-care, the “how” remains obscure. The chasm between successful research studies and actual introduction and scale up of innovations in routine settings is deep and wide (11). This study answers the WHO's recent call for “a stronger evidence base to promote the introduction, use and scale-up of self-care interventions (12).” If the promise of reproductive self-care is to be realized, we need program evaluations that build our understanding of how self-care can function successfully as an extension of the health system (8) and how self-care programs can be implemented in practice, elucidating and informing management of the repercussions–both good and bad–for women who participate in those programs.

Contraceptive self-injection (SI) is a new self-care practice with the potential to transform women's family planning access and reproductive autonomy by putting a popular method–subcutaneously-administered injectable contraceptive depo medroxyprogesterone acetate (DMPA-SC)-directly into the hands of users. Women who self-inject at home save money and time and are less subject to the vagaries of a public health system frequently compromised by commodity stock outs, health worker absenteeism, and restricted access to family planning services (13–15). There is considerable evidence from research studies that self-injection is feasible and acceptable to women (16–18), and that self-injection can help women to continue uninterrupted injectable use (19–22). However, it can be challenging to replicate results obtained in a carefully controlled research settings in the context of routine public sector service delivery. Evidence and guidance regarding *how* SI programs can be designed and implemented at scale under real-world conditions is very much still needed (23).

To this end and in partnership with the Ugandan Ministry of Health (MOH), PATH–a global nonprofit that advances health and well-being in low resource settings–developed, implemented and evaluated a pilot program to generate evidence and guidance to design and implement SI programs in low resource settings. The Uganda National Drug Authority granted approval for DMPA-SC for self-injection in 2017. Subsequently, under the Self-injection Best Practices Project, PATH undertook a human centered design approach to engage clients, providers and MOH stakeholders in developing an SI pilot program to be implemented at public sector health facilities in select districts (24). Lessons from this program would ultimately inform the development of the MOH Self-injection Guidelines. With training from PATH, providers–both clinic and community based-began offering SI as an option for women in 2018. Self-injection was provided as one of the available voluntary contraceptive options at participating service delivery points. Over the first year of implementation, more than 4,000 women opted for self-injection under this pilot program.

The goal of the evaluation, conducted in 2019, was to identify programmatic approaches to SI service delivery in the public sector that offer the greatest promise of producing successful self-injectors in a way that is feasible and efficient. We also assessed longer-term outcomes, including how long women continued with self-injection, the extent of product wasted when women stopped self-injecting, and how women disposed of used devices. Finally, we investigated why some women opted not to self-inject despite receiving training.

## Materials and methods

### Program description

Through the Self-Injection Best Practices project, more than 8,000 women in three pilot districts (Gulu, Mayuge and Oyam) received training in SI over the course of 1 year, representing approximately one quarter of family planning visits at the participating service delivery points. Self-injection program service delivery guidelines stipulated that health workers introduce SI in the context of informed choice (one of many contraceptive options), and for women who were interested, that they cover five key topics during training: when and how to reinject, how to securely store devices, how to safely dispose of used units, and what to do in the event of problems. It was left to health workers to decide whether to train clients individually or in a group, since workload and client flow on any given day would likely dictate training format. However, when conducting a group training, health workers were advised to limit group size to no more than 10 individuals. Health workers were instructed to demonstrate the injection technique during training; to train clients in how to use the job aid (instruction sheet) and how to calculate reinjection dates; and to provide a job aid and calendar as memory aids. Under the service delivery guidelines, they could give women two units to take home following their initial, successful self-injection performed under health worker supervision. Clients were advised to return (or call) if they experienced challenges, and to return used units to the health worker (clinic- or community-based) at their convenience for safe disposal.

Beyond these common guidelines, a number of approaches to SI service delivery in the public sector were systematically varied across sites (defined as the clinic and its catchment area) in three districts^1^, such that some sites offered SI training services at the clinic and others in the community through community health workers; some sites encouraged women to practice injecting on a model, while others offered only health worker demonstration; some sites provided women with a hotline phone number to call for any post-training follow up needs, while others did not offer that service.^2^ A total of 34 sites were selected in a purposive fashion to capture variation in health system levels (Health Center II, III, and IV), and were randomly assigned to the different program models. In November and December of 2017, 97 clinic-based and 83 community-based health workers, called Village Health Teams (VHTs) were trained to offer self-injection services in the three districts.

### Evaluation objectives

The main objective of the evaluation was to assess the effectiveness and feasibility of different program designs for self-injection, focusing on injection competency as the key measure of effectiveness (e.g., a successful SI client and by extension, successful program approach). Additional objectives were to capture information about the nature and content of the training received, client satisfaction with training, any post-training follow up, and client experiences with independent SI. For a subset of those interviewed, the goals were to learn about waste disposal practices, experiences with resupply of DMPA-SC, and continuation with self-injection. We also interviewed a subset of women who participated in SI training but declined to self-inject to better understand their reluctance. The views of health workers regarding the feasibility and acceptability of the SI program and the experiences of adolescent self-injectors are reported elsewhere (25, 26).

### Evaluation design

We conducted structured survey interviews with a sample of SI clients who received training at clinics or from VHTs in Gulu, Mayuge, and Oyam districts from May to July 2019, ~4 months after their SI training. The survey included questions covering socio-demographic characteristics, contraceptive history, experience with SI services (training), and experiences with independent self-injection (reinjection). Survey questions were developed through an iterative and collaborative process that engaged the full research team, and pretested prior to initiating data collection.

The sample of participants was randomly drawn from monitoring data collected in SI training registers; in order to be eligible, women had to have participated in SI training, *and* either self-injected after training or taken units home (or both, as in most cases). Participants also had to be willing to be contacted at a later date, after their first scheduled reinjection, for an interview about their self-injection experience.

In addition to the interview, SI clients were asked to demonstrate their injection technique on a model and were evaluated using a nine-step observation checklist to measure injection proficiency. To be evaluated as proficient, clients must demonstrate mastery of the four injection steps: (1) mix the liquid by shaking the device, (2) activate the device to break the seal between the reservoir and the needle, (3) pinch the skin to form a “tent” for subcutaneous injection, (4) squeeze the reservoir slowly to inject. The injection proficiency rate is the percent that demonstrate mastery of these four critical steps. Clients were not given any guidance or instruction during their demonstration, but were encouraged to use the job aid, as needed.

We followed up with a subset of SI clients for a second interview between 10 and 17 months after their training. Half of these interviewees were from Gulu and half from Mayuge, with half trained by VHTs and half by facility-based health workers. Only clients who had self-injected independently at least once subsequent to training were eligible to participate in a follow-up interview. Survey questions for this interview focused on experiences with subsequent self-injection, resupply, waste disposal and reasons for and timing of discontinuation.

In addition, we interviewed a random sample of clients who were recorded in the training registers as having participated in SI training but declined to self-inject. To be eligible, “non-adopters” neither self-injected nor took units home at the end of their training. They also had to express willingness to be contacted at a later date for an interview. Interviews took place between May and July 2019, 10 to 17 months after training. This structured survey focused on socio-demographic background, contraceptive history, experiences receiving SI services and reasons for declining self-injection.

### Sample selection

For the structured SI client survey, a sample size of 960 women would permit detection of a 10 percentage point difference in injection proficiency measured at the time of the second injection (3+ months post-training) between programs that vary across sites, with statistical power of 80%. Eligible participants were stratified equally by district, then randomly selected (using the random sampling function in Stata) from a database (enumeration frame) compiled from the registries of clients who received SI training at one of the 34 participating sites and who had self-injected (or taken units home) after training, and who had indicated a willingness to be contacted. If a woman was not locatable (or declined to participate), the next randomly selected SI client from the same district replaced her.

For the follow up interviews with self-injectors and with non-adopters, a sample size of 200 from each group was considered sufficient for these supplemental exploratory analyses. Similar to sample selection of SI clients, non-adopters were stratified equally by district, then randomly selected from the SI training database.

### Data collection and analysis

Six female research assistants were trained in research ethics and the informed consent process, study procedures, interviewing techniques–both theory and practice, instrument review, observing and evaluating injection proficiency, use of electronic data collection tools and data management practices. They conducted face to face private interviews in English, Luganda, Acholi or Langi, depending on the participant's preference, with responses entered electronically on cell phones using Open Data Kit (ODK) software. The observation checklist to evaluate injection proficiency was first completed on paper, then entered electronically using ODK. Women were also asked about the timing of their first reinjection. Any self-injection given during the WHO-approved DMPA reinjection window (−2/+4 weeks) was considered to be on-time. For the second round of interviews with the subsample of SI clients, as well as the interviews with non-adopters, injection proficiency was not assessed.

Structured survey data were analyzed using Stata IC/14.2 software. Chi square and student *t*-tests were used to evaluate differences between groups, using conventional significance levels of 95% with two-sided tests. Because women receiving services in the same clinic or community are likely to share similar characteristics and are exposed to similar SI training approaches, the assumption of independence for individual women may not be valid. Therefore, the multivariate analysis predicting injection proficiency and self-injection adoption employed a mixed effects logistic regression, accounting for between cluster variability using the location of service (clinic) as the random effect. Fitting a standard logistic regression and mixed-effects logistic regression models, deviance (-2LLR), Akakie Information Criteria (AIC), and Bayesian Information Criteria (BIC) were used for model comparisons. The Adjusted Odds Ratios (AOR) with a 95% Confidence Interval (CI) and *p* ≤ 0.05 were used to identify statistically significant factors associated with injection proficiency and adoption of self-injection.

### Ethical considerations

All participants provided voluntary, written informed consent to participate in the evaluation. The study was approved by the Mulago Hospital Research and Ethics Committee and the Uganda National Council for Science and Technology (UNCST).

## Results

Out of a total of 4,340 women who became self-injectors in the public health sector in three districts, 958 (22%) were interviewed 4 months post self-injection training, after the window for their first reinjection closed. SI clients were trained between February and July 2018 and were interviewed from June to November 2018. A subsample of 200 SI clients were re-interviewed at least 10 months post-training, and 200 nonadopters were interviewed at least 7 months post-training, from May to July 2019.

### SI client characteristics

As a reflection of the representativeness of the SI clients who participated in the evaluation, Table 1 shows the basic background characteristics of survey participants compared with training register data for all SI clients (the enumeration frame). Evaluation participants had significantly more education on average than all SI clients (just 5% had never been to school vs. 14% among all SI clients). Participants were slightly older, with a greater share falling into the category of 25 years and up (57 vs. 52%), significantly less likely to be first time contraceptive users (24 vs. 29%) and significantly fewer had traveled for at least an hour to reach the facility or VHT (26 vs. 38%) as compared with all SI clients. These distinctions reflect differential willingness to participate in an interview and difficulty contacting or locating more remote participants. Though nearly all participants who were contacted by the evaluation team agreed to participate, not all clients listed in the registry were willing to be contacted, and therefore did not provide their contact information to the health worker when trained. In some cases, contact information was insufficient to track down a potential participant.

**Table 1:** Self-injection client background and family planning experience.

**Characteristic**	**Survey data**	**Registry data**
	**percent (*n*)**	**percent (*n*)**
	***N* = 958**	***N* = 4,340**
Education		
None	4.9% (47)	14.0% (609)*
Primary	70.8% (678)	NA
Secondary +	24.3% (233)	NA
Mean years of education	6.2 (958)	NA
Missing	0	0.5% (22)
Age		
<20 years	11.9% (114)	14.2% (618)
20–24 years	30.9% (296)	32.4% (1,406)
25+ years	57.2% (548)	52.3% (2,268)
Missing	0	1.1% (48)
Mean age	26.6 (958)	26.0 (4,292)^*^
Marital status		NA
Single or not in union	11.9% (114)	
Married or living together	88.1% (844)	
First time user of contraception	23.5% (225)	29.3% (1,273)^*^
Missing	0	0.5% (22)
First time user of injectable contraception	36.3% (351)	34.6% (1,502)
Missing	0	0.5% (22)
Last method used		NA
None/never used	23.5% (225)	
DMPA-IM	44.1% (422)	
DMPA-SC	14.7% (141)	
Implant	7.4% (71)	
Oral contraceptives	4.4% (42)	
Other (condom, IUD, LAM, cycle beads/abstinence)	5.9% (57)	
Distance traveled to reach FP services (either clinic or VHT)		
<30 min	35.3% (338)	NA
30 min to 1 h	38.4% (368)	NA
1 or more h	26.3% (252)	37.7% (1,635)^*^
Missing	0	1.6% (71)

* *Significant difference between the values indicated in each category at the p < 0.05 level*.

*NA, data not available. A limited set of measures were included in the register (e.g., women were asked yes or no whether they had attended school or traveled 1 h or more to reach the clinic)*.

*VHT, Village health team*.

### Self-injection proficiency

The proficiency rate for SI clients, defined as demonstrated mastery of the four critical steps–Mixing, Activating, Pinching and Slowly injecting-was 72.6%. If we limit the analysis to those who continued self-injecting after their training (e.g., reinjected at home), 75% were evaluated as proficient (not shown). The most common steps missed in the demonstration were activating the device and pinching the skin to create a tent.

Proficiency did not significantly differ for those trained by VHTs in the community (70%), as compared with those trained by clinic-based health workers (75%) (Table 2). Just over half of evaluation participants were trained for self-injection by a VHT in the community.

**Table 2:** Training experiences and association with injection proficiency, SI clients.

	**Percent (*n*)**	**Percent**
	***N* = 958**	**proficient**
Training context		
Clinic-based	48.0% (460)	75.2%
Community-based (by a VHT)	52.0% (498)	70.3%
Training format		
Group (only)	35.9% (344)	74.7%
One-on-one (only)	58.7% (562)	70.3%^*^
Both group and one-on-one training	5.4% (52)	84.6%^*^
Training techniques		
Practice	66.1% (633)	73.1%
Demonstration, no practice	19.7% (189)	74.6%
Neither demonstration nor practice	14.2% (136)	67.7%
Used job aid while training	96.6% (925)	73.4%^*^
Trained client to use/understand job aid	95.9% (913)	73.7%^*^
Provided job aid to take home	89.4% (856)	75.1%^*^
Training content		
Training included all 5 key topics+	82.4% (789)	76.1%^*^
One or more topics missed	17.6% (169)	56.8%^*^
Training duration		
<20 min	12.9% (124)	59.7%^*^
21 to 40 min	42.5% (407)	71.7%^*^
41 to 60 min	38.0% (364)	78.3%^*^
>60 min	6.6% (63)	71.4%^*^
Satisfaction with training		
Very satisfied	88.9% (852)	75.7%^*^
Somewhat satisfied	9.1% (87)	49.4%^*^
Somewhat unsatisfied	1.8% (17)	41.2%^*^
Very unsatisfied	0.2% (2)	50.0%^*^

* *Significant difference between the values indicated in each category at the p < 0.05 level*.

*+How to inject, when to inject, storage, disposal, what to do for problems*.

As per the evaluation design, sites were randomized to offer practice (or not), but about two thirds of clients reported practicing before self-injecting, suggesting that intervention fidelity was imperfect as health workers were not always faithful to those instructions (Table 2). Similarly, all health workers were expected to demonstrate the injection technique, yet about 15% of clients reported that they did not see a demonstration. Fundamentally, those who did not practice but saw a demonstration were equally proficient as those who practiced. Women who practiced multiple times did not exhibit greater injection proficiency (not shown).

Over one-third of clients were trained in a group setting (Table 2), with a median group size of 4 (not shown). The guidance to limit group size to 10 was not always followed, as 11% of group-trained clients reported being in a group with more than 10 individuals [up to a maximum of 50 (not shown)]. Group training was more common at clinics, while one-on-one training was more common among clients trained by VHTs in community settings (not shown). Just 5% of clients reported that they received one-on-one instruction in addition to group training, but these clients were significantly more likely than those who trained one-on-one (only) to demonstrate competency; their proficiency was not significantly different from those trained in a group only (not shown).

Nearly all clients (96.6%) were trained using the client instruction sheet or job aid, were guided in how to interpret the job aid (95.9%) and were given one to take home (93.7%). Familiarity with the job aid and being given one to take home were significantly associated with injection proficiency (Table 2); about three-quarters of women who had taken a job aid home or been exposed to one during training demonstrated proficiency, compared with one-third to one-half of those not exposed, and all these differences were highly significant (*p* < 0.01).

Eighty two percent of clients reported that their training covered five key topics (Table 2), with > 95% of clients indicating they were trained in how to inject, when to reinject, how to dispose and how to store, and > 90% were instructed in what to do if problems. Women whose training did not cover the five key self-injection topics were significantly less able to demonstrate injection proficiency, with approximately 76% competent if they received full training vs. 57% if they had not.

The median duration of training was 40 min (not shown). Training time was significantly associated with demonstrated proficiency, with shorter duration associated with lower proficiency (Table 2).

Proficiency varied significantly by education but not by other individual characteristics measured. On average, women who demonstrated injection proficiency had 6.4 years of education, vs. 5.8 years for those not competent (*p* < 0.05) (not shown). Overall, adolescent women under the age of 20 represented just 12% of the sample, but these young women were just as proficient as older women (not shown). Women new to family planning or new to injectable contraception were as proficient as experienced users (not shown).

In the multivariate mixed effects logistic regression (Table 3), years of education, having a complete training, taking home the job aid, and practicing the injection were associated with higher odds of demonstrating injection proficiency. The strongest effect was for those given a job aid, who had twice the odds of demonstrating proficiency (as those without). The effect of education while significant, was not large; each additional year of education was associated with < 10% greater odds of proficiency. It is notable that whether or not a woman had ever attended school was not a significant predictor of injection proficiency (not shown). Whether the training was conducted by a VHT in the community (or not) and whether only group training was provided were not associated with injection proficiency.

**Table 3:** Mixed effects multivariate logistic regression of injection proficiency.

**Covariate**	**Adjusted odds ratio**	**95% confidence interval**	***p*-value**
Education (years of)	1.08	1.02–1.14	0.01
Complete training	1.79	1.20–2.66	0.00
Provided with job aid to take home	2.00	1.23–3.24	0.01
Practiced injecting	1.50	1.01–2.21	0.04
Group only training	1.29	0.91–1.83	0.15
Trained by community VHT	0.81	0.40–1.61	0.54

### Independent self-injection

**Figure 1:**
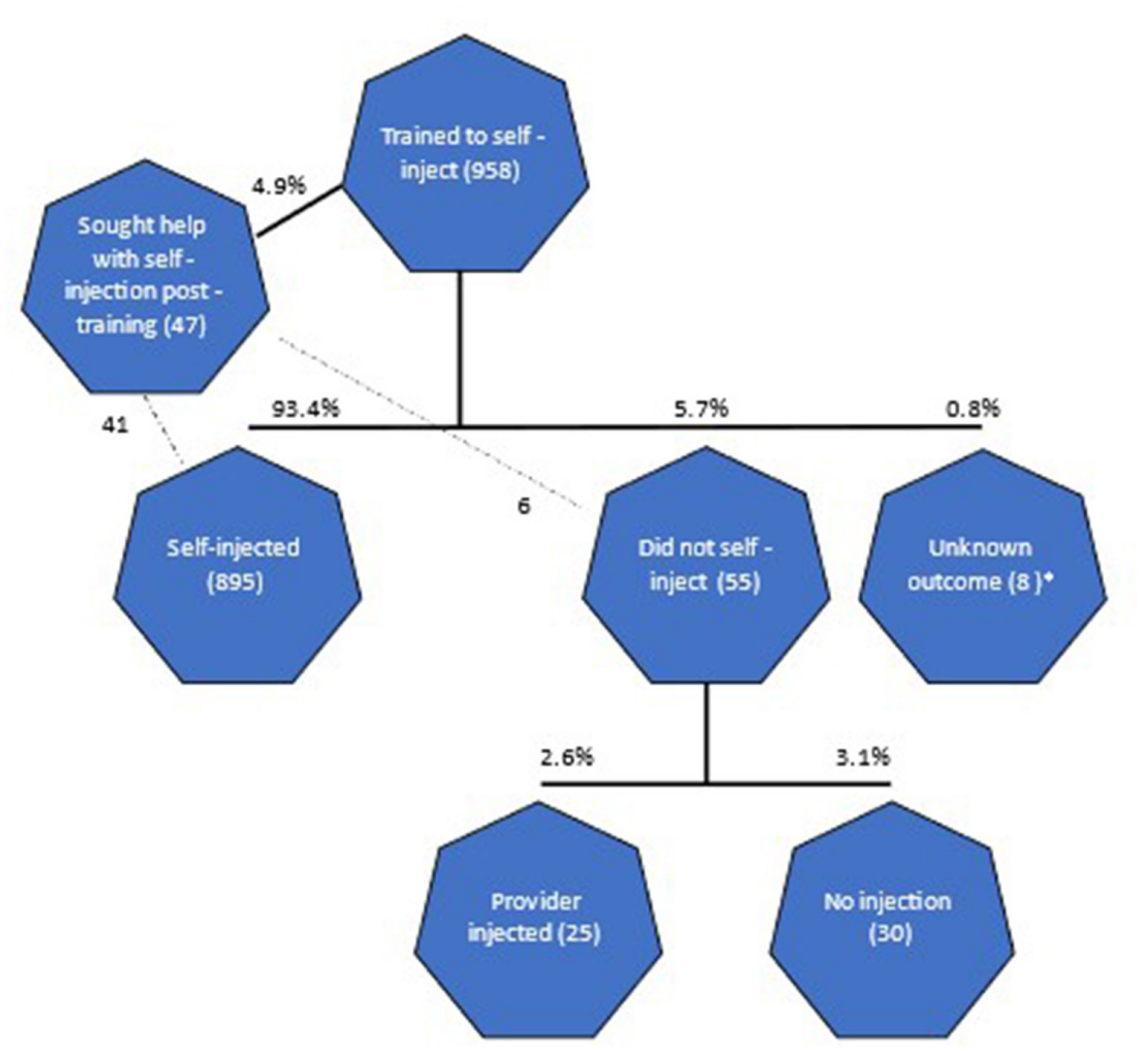
Flow diagram from training through subsequent contraceptive outcome. *8 individuals have conflicting information as they reported having self-injected yet also indicated zero times self-injecting.

Of the 958 SI clients, 93% (all but 63) went on to self-inject independently at home (Figure 1). Prior to self-injecting, 41 clients (5%) reported that they returned to the health worker for additional assistance with self-injection, and all but six of those proceeded with self-injection when due. Seven percent did not self-inject, including 30 (3%) who discontinued the injectable, 25 (3%) who reverted to health worker administration, and eight with missing data (<1%).

Self-reported injection proficiency was considerably higher than demonstrated proficiency, with 94% “very confident” they self-injected correctly, and 86% evaluating it as “very easy” to do (not shown).

Among those who reinjected, 93% did so on time, consistent with the WHO schedule. On-time reinjection was more likely if women were given a calendar and trained to use it: 94% of those trained in how to calculate their reinjection date and given a calendar to take home reinjected on time vs. 85% of those not trained and 86% of those not provided with a calendar (*p* < 0.05; not shown). Education was not significantly associated with on-time reinjection.

From a standpoint of product wastage, a total of 1,859 units were given to participants to take home for independent self-injection, of which 62 units (3%) may have gone unused, as the participant had not given or received an injection since training (at the time of the second injection).

### Continuation of self-injection

While continuation the second self-injection (first reinjection) was high (at 93%), of perhaps greater interest is continuation over a longer period. Our second interviews with a random sub sample of 200 SI clients, conducted 14 months after training (range from 10 to 17 months), revealed that 69% were continuing self-injection (Table 4). Four out of five in the follow up sample (80%) had continued self-injecting at least through their 4^th^ injection (having returned for resupply in the interim between interviews), which is the equivalent of a full year of contraceptive coverage (not shown).^3^

**Table 4:** Continuation and reasons for discontinuation of self-injection.

	**At 1st interview**	**At 2nd interview**
	**(4 mos post training)**	**(14 mos post training)**
	***N* = 958**	***N* = 200**
Current status		
Continuing self-injection at time of interview	93.4% (895)	69.0% (138)
Discontinued self-injection by time of interview	5.7% (55)	31.0% (62)
Unknown status, provided conflicting information	0.8% (8)	-
Reasons for discontinuing self-injection	*N* = 55	*N* = 62
Stopped DMPA-SC/wants child or pregnant^#^	9.1% (5)	35.4% (22)
Stopped DMPA-SC/method reasons (side effects, etc)	25.5% (14)	17.7% (11)
Stock out/No units given	10.9% (6)	17.7% (11)
Infrequent sex / partner away	(0)	12.9% (8)
Husband opposition	9.1% (5)	6.5% (4)
Health worker wouldn't permit self-injection	(0)	6.5% (4)
Challenges with self-injection	41.8% (23)	4.8% (3)
Other reason	3.6% (2)	(0)

# *It is likely (but not confirmed) that the five women pregnant at first interview were pregnant at the time of self-injection training; pregnancy testing when initiating FP is not routine. One individual had discontinued due to pregnancy by the time of the 2nd interview*.

At the time of the initial interview 4 months after training, difficulty self-injecting was the most common reason for not self-injecting independently, experienced by 42% of the 63 discontinuers (Table 4). Over time, as reflected in the follow-up sub-sample, challenges with self-injection became the least common rationale for stopping, whereas wanting a child became prominent, expressed by over one third of discontinuers. Method-specific reasons, such as side effects, were relatively common at both points in time. Challenges with obtaining units were also relatively common, particularly at resupply visits, where 18% of discontinuers noted they stopped self-injecting due to a stock out.

Among the 200 clients that participated in the follow up interview, 166 (83%) had returned for resupply, with more participants visiting a VHT in the community (61%) than a facility (39%). Almost all VHT-trained clients (97%) returned to their VHT, while about 25% of facility-trained clients visited a VHT for resupply (not shown).

**Figure 2:**
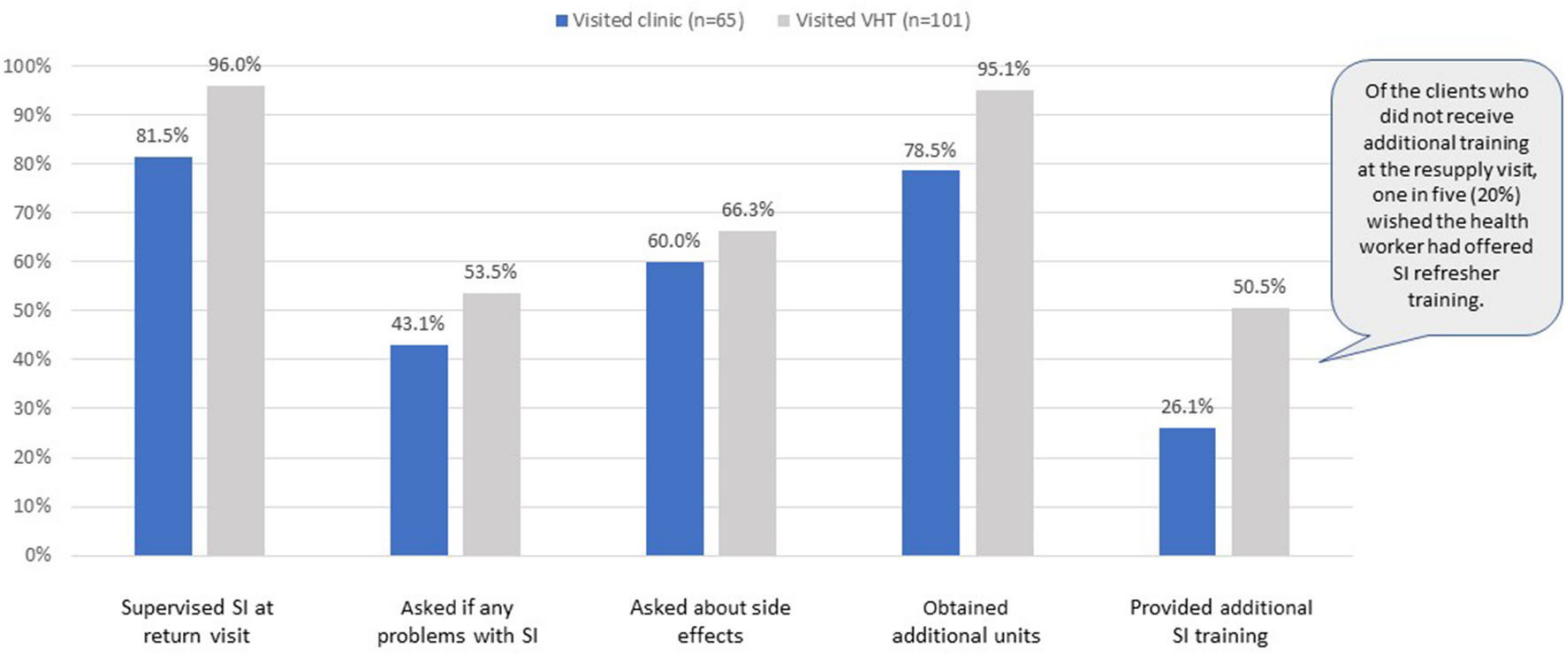
Experience at resupply visit.

There were significant differences in clients' resupply experience, depending on where they went for resupply. As shown in Figure 2, those visiting VHTs were significantly more likely to self-inject under supervision during their visit, obtain additional units, and receive additional training. Of the clients who did not receive additional training from either a VHT or clinic, approximately 20% wished they had.

### Disposal practices

**Figure 3:**
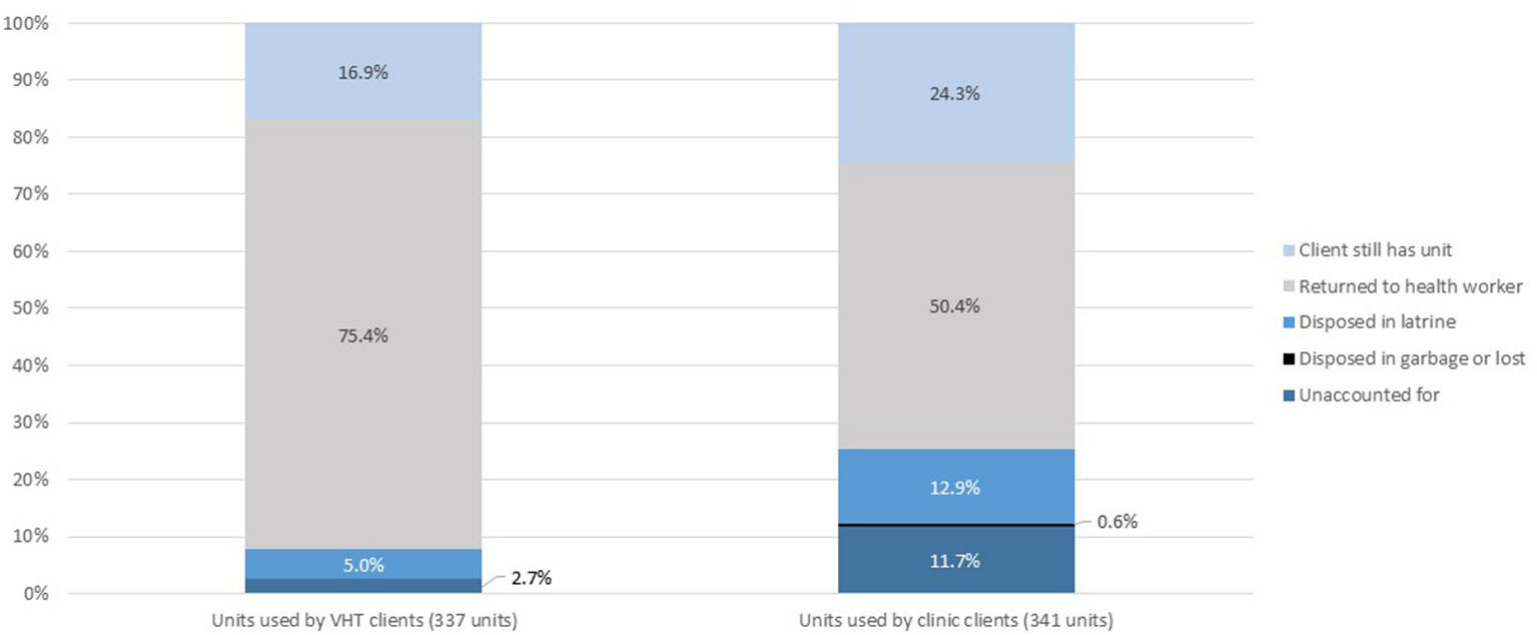
Distribution of used units, by training location (clinic or community).

Clients of VHTs also had different patterns for disposal of used units as compared with clinic clients. As shown in Figure 3, out of the 678 units used by participants in the follow up survey, units given to VHT clients were more likely to be returned to the health worker after use (75% of used units returned), consistent with service delivery guidelines, as compared with units given out to clinic clients (50% of used units). Overall, about 20% of spent units were still in the possession of clients, as they were waiting for the next resupply visit to return units.

**Figure 4:**
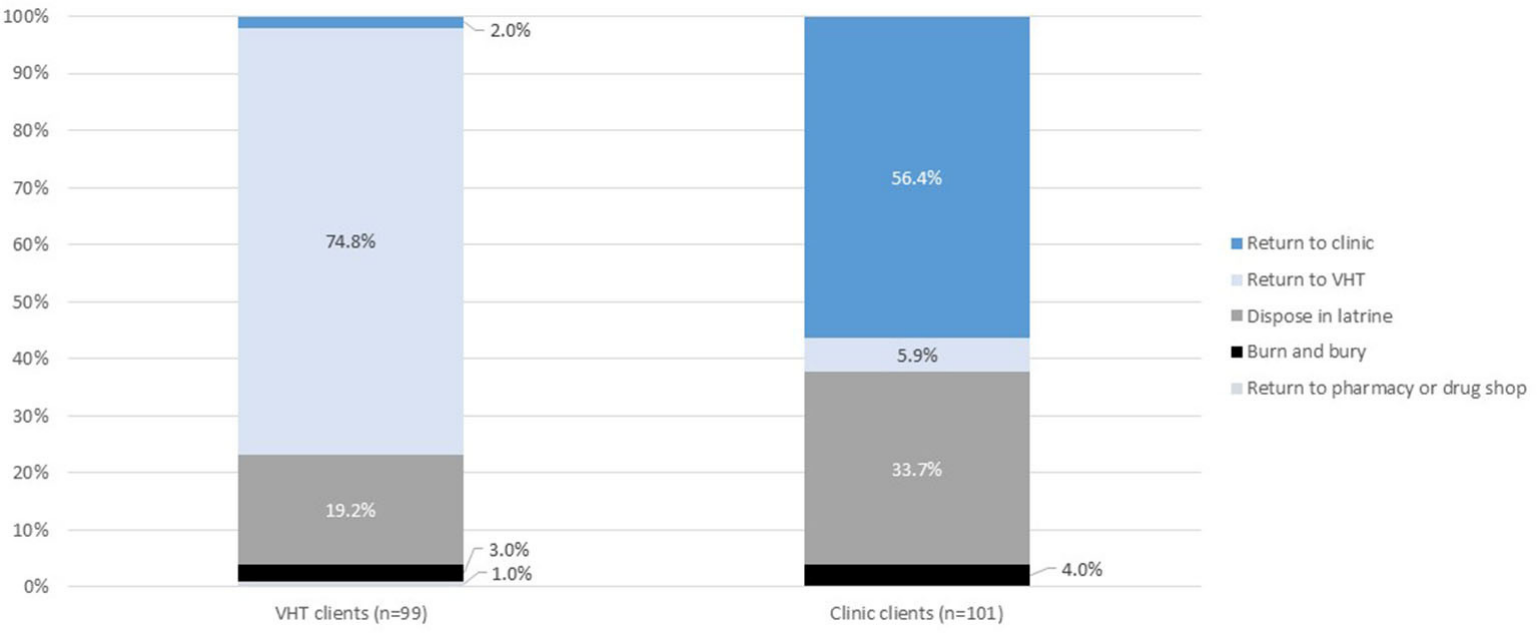
Disposal preferences by training location (clinic or community).

Turning to client preferences for disposal (Figure 4), clients generally preferred to return used devices to the place where they were trained (clinic or VHT). Clinic clients showed a stronger preference for latrine disposal relative to VHT clients (34% vs. 19%).

### “Non-adopter” clients

Among the more than 8,000 women who participated in a self-injection training, over 4,500 chose not to adopt the practice. Interviews with a sample of 200 of these “non-adopter” clients revealed that fear of injecting and/or lack of confidence was the most common reason for not self-injecting, expressed by about 90% (not shown). Nearly all the non-adopters ultimately received the injection from the health worker.

A multivariate mixed effects logistic regression reveals a number of ways in which SI clients differ from non-adopters, including both individual characteristics and characteristics of their SI training. As shown in Table 5, women whose partners are supportive of FP use and single women had higher odds of taking up self-injection. Training with a job aid, practicing the injection, witnessing a provider demonstration and exposure to a full training (covering all five recommended topics) were associated with higher odds of becoming a self-injection client; conversely, those trained in a group (rather than one-on-one) had reduced odds of taking up self-injection. Note that non-adopters did not differ significantly from self-injectors by age, education level, first time method users, first-time injectable users or whether trained by a VHT in the community or a clinic health worker (not shown).

**Table 5:** Mixed effects multivariate logistic regression of whether self-injection client.

**Covariate**	**Adjusted odds ratio**	**95% confidence interval**	***p*-value**
Trained with job aid	3.27	1.06–10.1	0.04
Practiced injecting	3.69	2.06–6.60	0.00
Provider demonstrated injection	3.26	1.58–6.72	0.00
Complete training	4.68	2.58–8.45	0.00
Trained only in a group	0.44	0.27–0.72	0.00
Single	5.44	2.14–13.84	0.00
Husband supports FP use	1.89	1.16–3.08	0.01

Some women who were trained may not have been particularly interested in self-injecting. For nearly one-third, it was the health worker who decided the client should participate in self-injection training (not shown). About 10% were visiting the health worker for a service other than family planning, and slightly fewer than half reported that they were “very motivated” to learn self-injection (not shown).

Among nonadopters, 23% ultimately returned for additional training, and 14% adopted self-injection in the interval between their original training date and the date of their interview.

## Discussion

This evaluation of self-injection service delivery under routine conditions found that the program was successful for the majority of women who opted to self-inject post-training, enabling most of the women who became SI clients to demonstrate self-injection proficiency. Nearly all self-injection clients who participated in the evaluation reinjected independently and the majority continued self-injection for at least 1 year. High rates of continuation contribute to low rates of product wastage. For the roughly one quarter judged not proficient in the admittedly artificial scenario of a demonstrated injection, it is difficult to know whether they successfully self-injected in real life. It is encouraging that they expressed confidence that they had self-injected successfully and about half of the small number not competent struggled with activation of the device, but were able to overcome that challenge to self-inject^4^.

Injection proficiency was not sensitive to training format–whether group or individual–which suggests health workers may adapt the SI training approach to accommodate their workload. This will be an important accommodation given the reported length of time required for training. However, one-on-one counseling exemplifies a central tenant of the WHO's framework for Quality of Care based on Human Rights Principles–respect for users' privacy and confidentiality (27). In other words, group counseling should always be coupled with individual attention to enable health workers to assess women's unique needs and counsel appropriately (28). Individual attention at some point in the counseling process remains important to aid clients who lack confidence, and fundamentally, for quality of care. A 2019 WHO-convened expert think tank identified “collaborative and confidential decision-making” as a key component of their definition of contraceptive counseling (29).

Proficiency was also not impacted by the type of trainer–clinic or community-based–echoing results from research in Malawi (10), where the majority of self-injectors were trained by community health workers. Our results show that community health workers are an important resource for self-injection programs. Not only were their clients equally competent, but they were competent despite lower education levels on average. According to their clients, VHTs were adept at resupply visits, being more likely to supervise self-injection, offer additional training and provide units for resupply. VHTs were also important resources for convenient, safe disposal in the community. Notably, our companion research on health worker perspectives on the SI program found that VHTs reported higher satisfaction and felt better able to accommodate self-injection training among the services they provide (18).

Injection proficiency is sensitive to training quality, with those who receive the job aid and whose training was comprehensive exhibiting greater proficiency. Lack of fidelity to program guidelines for the training approach and content–not using or distributing the job aid, group training that exceeds the recommended size, lack of demonstration, abbreviated training–highlights the importance of post-training supportive supervision and monitoring to ensure that providers are able to deliver high quality SI programming.

While the effect of education was not pronounced, those with more education were better able to demonstrate proficiency. This suggests that women with less education may benefit from more support to master the injection technique. While not measured directly, limited literacy likely impacts their ability to navigate the job aid independently post-training, necessitating greater guidance (including training in how to interpret the job aid). Regardless of education level, women are competent to manage their own injection with appropriate training and support. The finding that women without any education were *as competent as those who had attended school* should reassure stakeholders that successful self-injection does not require schooling. This is an important “non effect” since women without education may face discrimination in accessing self-injection services because they are perceived to be not appropriate for self-care, despite standing to benefit the most because of barriers they face in accessing SRH information and services (30). Our findings reiterate the WHO focus on health literacy and information as key enabling factors for successful self-care (7). In the same vein, this study, and that of a companion piece focused on the adolescent experience (19) found that adolescents are equally proficient at self-injection as older women; this is an important “non effect” as they too may face discrimination in accessing self-care products and services.

Turning to the substantial number of individuals who trained but opted not to self-inject, our non-adopter findings should prompt reflection on how programs can focus on the women most likely to find the innovation compelling and provide an optimal training approach that builds self-efficacy for self-injection. With respect to the non-adopter phenomenon, Diffusion of Innovations Theory suggests five factors that inform how we think about the adoption of a health innovation: (1) the perceived relative advantages in terms of personal, physical, social and/or economic benefit; (2) compatibility of the innovation with felt needs and sociocultural values; (3) trialability or testing the innovation before committing; (4) the complexity of the innovation; and (5) observability or witnessing others experiencing benefits of the innovation (31, 32).

Viewed through the Diffusion Theory lens, we can envision multiple explanations for the “non adopter” phenomenon. First, self-injection training may be directed to women who may be more curious than motivated–that is, they may not see the relative advantages or may not see self-injection as addressing a felt need. Health workers excited by a new option may be inclined to try to “convert” to self-injection women who would ultimately prefer to receive the injection from a health worker. It is also possible that health workers felt motivated to shift women into self-care approaches in anticipation of reducing subsequent visits and consequently, their workload. Rights-based family planning service delivery requires that, as self-care interventions and methods are rolled out, principles of informed choice emphasize not just method choice, but mode of delivery, whether by a health worker or self-initiated. As self-injection moves to scale, program managers should anticipate that health workers will require training in the elements of rights-based service delivery, including person-centered care (33), and specifically, counseling that gives full weight to client method *and delivery* preferences. At the same time, communication and counseling around self-injection should clearly address the potential benefits, including the personal and economic benefits of saving time and travel costs.

Second, while self-injection is not complicated, its perceived complexity will depend on the quality of the training provided. A full self-injection training requires time: 35 to 60 min for the average participant as reported by clients, and 20 to 40 min as reported by health workers (18). When training is abbreviated, and does not include critical information on how and when to inject, storage, disposal and what to do if there are problems, as well as review of the job aid, demonstration or practice, the perceived complexity of self-injection increases, and self-injection is seen as personally risky, with uncertain outcomes (34). Under these conditions, even women who are motivated and see the relative advantage of self-injection may lack the confidence to self-inject.

Third, some women will want to try out self-injection before committing. Particularly for women with injection anxiety, adopting self-injection may be a process that requires repeat exposure to the concept and injection technique, and could be facilitated by more intensive support from health workers. Again, consistent with diffusion theory, clients may move through the “innovation-decision process” from knowledge, persuasion, decision, implementation, and confirmation to arrive at adoption at different speeds and requiring different support (21). Our results suggest that clients who are fearful may benefit from practicing with the device and one-on-one training with a health worker to overcome fears and gain the confidence to self-inject. Finally, our study suggests that health workers may need [and would welcome (18)] additional support in how to counsel women with injection anxiety effectively, keeping in mind that self-injection must always be a client choice, not a requirement.

Finally, the findings that single women, and those with partners supportive of family planning are more likely to become self-injection clients remind us of the role of autonomy and agency in adoption of self-care methods. While self-care is often touted as a means of advancing women's self-determination, self-efficacy and autonomy (7), women who already possess agency and autonomy appear to be better able to take advantage of self-care services. Further research is needed to better understand how we can structure programs and services to provide greater access to self-care for all women, paying particular attention to those with limited agency and autonomy.

### Study limitations

As is the case with most social science research, interview responses are likely shaped to an unknown degree by social desirability bias and courtesy bias, translating into overly positive reflections on the training received and their self-injection experience. In the case of the non-adopter findings, recall bias is also possible, particularly with respect to descriptions of the training, given the length of time since their training. (Respondents were given the opportunity to say “do not recall” and the small number of such responses were excluded from analysis.) Self-injection was not observed in this study, with a simulated injection serving as a proxy measure of injection proficiency. The experience of demonstrating the injection may have made some participants nervous or may have been taken less seriously by some participants relative to the care they would take for an actual self-injection. In either case, this could result in lower proficiency scores.

## Conclusion

This study, the first to evaluate a self-injection program under routine conditions, provides evidence for ministries of health and program managers to consider as they develop guidelines for self-injection programs. Specifically, our results emphasize the vital role that community health workers may play not only in raising awareness of self-injection but as self-injection trainers; the feasibility of group training–with appropriate group size limitations–as an option in busy clinic settings, the importance of job aids for home use, and the value of a comprehensive training.

## Data availability statement

The raw data supporting the conclusions of this article will be made available by the authors, without undue reservation.

## Ethics statement

The studies involving human participants were reviewed and approved by Mulago Hospital Research and Ethics Committee and the Uganda National Council for Science and Technology (UNCST). The participants provided their written informed consent to participate in this study.

## Author contributions

JC, AN, CM, JT, and DN develop the protocol and study materials. AN, JT, and DN trained research staff and oversaw study implementation in the field. JC and CM conducted the analysis. JC, AN, CM, JT, DN, and JD contributed to drafting the manuscript. All authors contributed to the article and approved the submitted version.

## Funding

This work was supported by the Bill & Melinda Gates Foundation, Seattle, WA Under Grant Number: 1154309. The funding source did not play a role in study design; the collection, analysis, and interpretation of data; the writing of the report; or the decision to submit the article for publication.

## Conflict of interest

Authors JC, AN, JT, JD, CM, and DN were employed by PATH.

## Publisher's note

All claims expressed in this article are solely those of the authors and do not necessarily represent those of their affiliated organizations, or those of the publisher, the editors and the reviewers. Any product that may be evaluated in this article, or claim that may be made by its manufacturer, is not guaranteed or endorsed by the publisher.

## Footnotes

^1^In a fourth district (Mubende), the program was rolled out through an adolescent service delivery platform, involving 10 facilities and affiliated safe spaces. Adolescent experiences with the program are reported elsewhere (19).

^2^While a random sample of facilities were assigned to the “hotline follow up” approach, only three women surveyed reported calling the hotline and all three sought side effects advice.

^3^An important caveat is that only those who had self-injected at least once since training were eligible for the 2^nd^ interview. Therefore, it would not be accurate to conclude that 80% of *all* self-injectors continued through four injections, but rather 80% *of those who self-injected at least once post-training* continued through four injections. Six individuals reported they were continuing with self-injection but had not self-injected four times, suggesting a gap in contraceptive coverage. They are not counted among the 80%.

^4^Activating the device breaks the seal between the reservoir and the needle, thereby releasing the product; without activation, the product cannot be expelled.
